# Relative contributions of vegetation and soil properties to microbial community structure and function in alpine and subalpine meadows of the southeastern Tibetan Plateau

**DOI:** 10.3389/fmicb.2026.1847498

**Published:** 2026-06-08

**Authors:** Zheng Hou, Minnuo Shi, Shuai Gou, Danqi Liao, Changxing Hu, Qiuyu Zhang, Xinyue Zhang, Liting He, Yong Ba, Ya Zhang, Yuanlong Li, Kaixuan Zhou, Hu Wang, Lin Song

**Affiliations:** 1 Kunming General Survey of Natural Resources Center, China Geological Survey, Kunming, China; 2 Technology Innovation Center for Natural Ecosystem Carbon Sink, Ministry of Natural Resources, Kunming, China; 3 Innovation Base for Eco-Geological Evolution, Protection and Restoration of Southwest Mountainous Areas, Geological Society of China, Kunming, China; 4 School of Ecology and Environmental Sciences, Yunnan University, Kunming, China; 5 Ecology and Environment Bureau of Diqing Tibetan Autonomous Prefecture, Shangri-La, China; 6 Runziyuan Environmental Protection Technology (Yunnan) Co., Ltd., Kunming, China

**Keywords:** alpine meadow, microbial community, microbial function, subalpine meadow, Tibetan Plateau

## Abstract

**Introduction:**

Ongoing climate warming is expected to promote the upward expansion of subalpine meadows and the gradual replacement of alpine meadows on the southeastern margin of the Tibetan Plateau. However, the mechanisms by which these vegetation transitions reshape belowground microbial taxonomic composition and metabolic functional potential remain poorly understood.

**Methods:**

We investigated soil microbial community structure and functional potential in alpine meadow (AM) and subalpine meadow (SM) ecosystems in the Napahai Basin by integrating vegetation surveys, soil chemical analyses, enzyme activity assays, and metagenomic sequencing.

**Results and discussion:**

Altitudinal differences in hydrothermal conditions were associated with pronounced divergence in plant community composition and soil nutrient status between the two meadow types. Although microbial α-diversity did not differ significantly, β-diversity analyses revealed distinct taxonomic and functional differentiation. Functional annotations based on CAZymes and KEGG indicated that variation in microbial functional potential was closely associated with coordinated changes in carbon, nitrogen, and phosphorus availability, suggesting that microbial metabolic strategies shifted along the environmental gradient. Random forest and partial least squares path modelling further showed that plant community composition exerted a stronger direct influence on microbial functional configuration than soil-mediated indirect effects. These findings highlight the prominent role of vegetation in shaping microbial functional potential and underscore the sensitivity of belowground ecological processes to vegetation transitions along environmental gradients in high-elevation meadow ecosystems.

## 1 Introduction

The Tibetan Plateau, with an average elevation of approximately 4,300 m and an area of about 2.5 million km^2^, is one of the highest and largest plateau ecosystems in the world. Nearly 70% of its surface is covered by various meadow types (Zhu et al., 2023). These meadow ecosystems provide essential ecological functions, including water conservation, soil retention, biodiversity maintenance, and carbon sequestration, and are therefore regarded as important ecological security barriers (Fu et al., 2021). However, under ongoing global climate warming, the Tibetan Plateau has warmed at a rate of 0.42 °C per decade over the past four decades, approximately twice the global average (Yao et al., 2022). As one of the most fragile alpine regions worldwide, the structure and function of plateau meadows can be strongly affected by even slight climatic or anthropogenic disturbances (Zhu et al., 2023). In this context, soil, as the foundation of meadow ecosystems, supports plant growth and microbial activity and plays a key role in maintaining ecosystem resilience (Li et al., 2020). Consequently, soil processes in alpine meadow ecosystems have received increasing research attention. Soil microorganisms are widely recognized as important biological indicators of soil quality (Wang D. et al., 2022). As major biotic components linking plant-derived inputs with soil nutrient transformation, they regulate material cycling and energy flow in terrestrial ecosystems. Because soil microbes can respond rapidly to environmental change (Xiao et al., 2024), they are considered pivotal to grassland succession and ecosystem service provision. Recent studies have further shown that soil microorganisms can enhance the resilience of plant–soil systems by maintaining network stability (Du et al., 2025), highlighting their importance in grassland restoration.

In recent years, studies of soil microorganisms in meadow ecosystems have focused primarily on degraded grasslands. Numerous studies have shown that nutrient loss associated with grassland degradation reduces soil microbial richness and decreases the abundance of microbial taxa that promote plant growth (Liu K. et al., 2023). Degradation-induced changes in soil nutrient availability can strongly affect bacterial and fungal diversity (Wu et al., 2021), reduce the complexity of microbial co-occurrence networks (Qiu et al., 2021), and ultimately alter community structure and functional traits (Cao et al., 2021; Liu K. et al., 2023). At the functional level, a small but growing body of evidence suggests that soil substrate availability and environmental conditions are key regulators of microbial carbohydrate-active enzyme (CAZyme) profiles. Grassland degradation is often accompanied by reduced litter inputs, which may weaken the CAZyme gene pool involved in complex carbohydrate degradation and constrain the capacity of microbial communities to utilize complex carbon sources (Zhang et al., 2023). However, the Tibetan Plateau hosts diverse meadow ecosystem types that exhibit pronounced spatial differentiation along altitudinal gradients. Among them, alpine meadows (AM) and subalpine meadows (SM) are adjacent and transitional meadow types that are particularly sensitive to climate change (Liu M. et al., 2023; Xu and Du, 2023). The Napahai Basin is a plateau fault-depression system characterised by high surrounding mountains and a low central basin, with a mean lake water level of approximately 3,263 m. In this region, alpine meadows are mainly restricted to the Shika Snow Mountain region on the western side of the basin above 3,700 m, where they experience lower temperatures, stronger winds, and shorter growing seasons; they are dominated by *Ligularia cymbulifera*. In contrast, subalpine meadows mainly occur below 3,700 m under relatively warm and moist conditions and are dominated by mesic perennial grasses and forbs, with *Euphorbia stracheyi* as the dominant species. Unlike grassland degradation, which is mainly driven by anthropogenic disturbance and is often associated with ecosystem decline, vegetation type shifts along altitudinal gradients are largely climate-sensitive processes reflecting long-term environmental filtering and biotic reassembly. These processes involve coordinated changes in plant community composition, soil microclimate, and resource inputs, thereby potentially inducing distinct microbial assembly and functional responses that cannot be fully inferred from degradation contexts. Building on extensive studies of grassland degradation, recent research has increasingly shifted toward climate-sensitive vegetation transitions along environmental gradients (Liu et al., 2024; Yin et al., 2024). However, compared with the well-documented effects of degradation on soil microbial communities, how climate warming drives the distributional shifts between alpine and subalpine meadows and subsequently influences soil microbial community structure and functional potential remains insufficiently understood.

Across the Tibetan Plateau, alpine and subalpine meadows differ systematically in temperature and moisture regimes, vegetation composition, and soil physicochemical properties. Under ongoing global warming, the upward expansion of subalpine meadows and their gradual replacement of alpine meadows at higher elevations have been widely predicted (Niu et al., 2019; Ma et al., 2024). This transition involves not only shifts in aboveground vegetation but may also reshape belowground microbial taxonomic composition and functional potential by altering plant-derived inputs and soil environmental conditions. However, compared with the extensive research on degraded grasslands, much less is known about how climate-associated transitions between adjacent meadow types influence soil microbial communities and their metabolic potential. Here, we investigated alpine and subalpine meadows in the Napahai Basin on the southeastern margin of the Tibetan Plateau. By combining vegetation surveys, soil physicochemical and enzymatic analyses, and metagenomic sequencing, we aimed to determine how soil microbial community composition and functional potential differ between meadow types and to identify the plant and soil factors underlying these differences. We hypothesized that (1) soil microbial community composition and functional potential differ significantly between alpine and subalpine meadows, and (2) meadow-type differences drive shifts in microbial taxonomic structure and functional potential through changes in vegetation composition and soil properties.

## 2 Materials and methods

### 2.1 Study area overview

**Figure 1:**
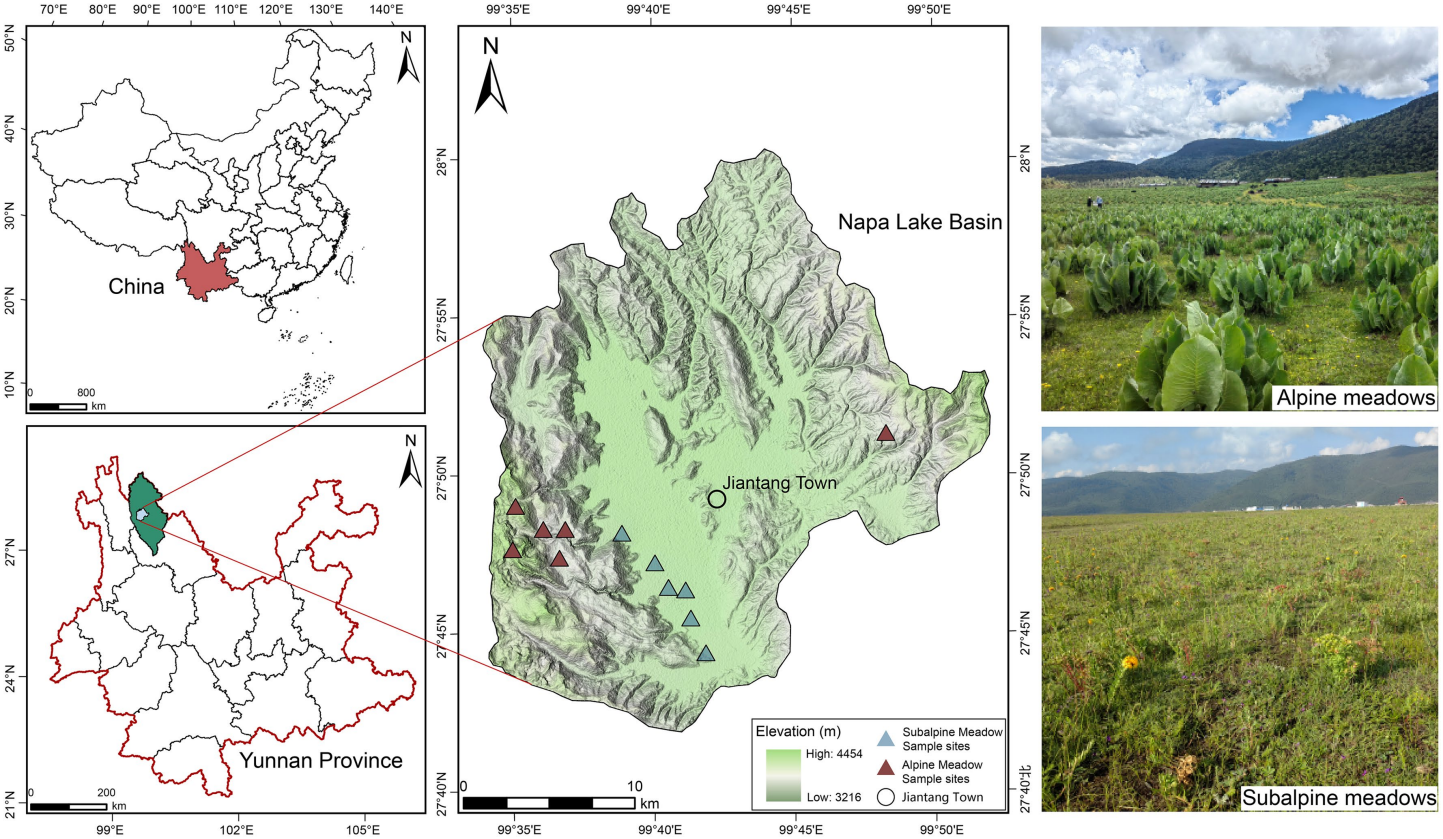
Study area location map (the review number: GS (2024) 0650). The map was created using ArcGIS Pro software (version 3.1.5) (https://www.esri.com/zh-cn/arcgis/products/arcgis-pro/overview).

The Napahai Basin is located in Shangri-La City, Diqing Tibetan Autonomous Prefecture, in the middle section of the Hengduan Mountains on the southeastern margin of the Tibetan Plateau (27°41′48″–28°00′15″N, 99°33′30″–99°51′46″E; Figure 1). The basin covers an area of 596.05 km^2^, with elevations ranging from 3,260 to 4,414 m. The region has a cold temperate monsoon climate with typical low-latitude plateau monsoon characteristics. The mean annual temperature is 5.4 °C, and the mean annual precipitation is 620 mm. Distinct dry (November–April) and wet (May–October) seasons occur, with large diurnal temperature variations, a long frost period, and strong solar radiation. The mean annual sunshine duration is 2180.3 h (Lu et al., 2019). Vegetation in the study area is dominated by alpine pine forest and alpine shrubland, alpine meadow, and subalpine meadow. According to the World Reference Base for Soil Resources (WRB), the AM sites were primarily developed on Haplic Cambisols, which are weakly to moderately developed mountain soils characterized by cambic horizon formation and relatively high organic matter accumulation under cold, high-elevation conditions. In contrast, the SM sites were mainly associated with Haplic Luvisols, which are more strongly developed and well-drained mineral soils characterized by clay-enriched subsurface horizons, clearer horizon differentiation, and higher pedogenic maturity. In the wider basin context, Umbric Gleysols are mainly distributed in wetter, low-lying meadow or lakeshore areas and are not representative of the soil backgrounds of the sampled AM and SM sites.

### 2.2 Plot design and sample collection

Alpine meadows and subalpine meadows in the Napahai Basin were selected as the study systems. Alpine meadow sites ranged in elevation from 3715.1 to 4333.56 m, whereas subalpine meadow sites ranged from 3264.2 to 3307.14 m. The dominant species were *Ligularia cymbulifera* (W.W. Smith) Hand.-Mazz. in alpine meadows and *Euphorbia stracheyi* Boiss. in subalpine meadows. Because the meadow patches in the study area were relatively flat and homogeneous, with low within-plot vegetation heterogeneity, the sampling design followed the Technical Regulations for National Forest, Grassland and Wetland Survey and Monitoring issued by the National Forestry and Grassland Administration of China. At each site, one circular plot of 0.5 ha (radius: 40 m) was established as the site-level sampling unit. Using the plot centre as the reference point, three 1 m × 1 m quadrats were placed near the plot margin along radial directions of 0°, 120°, and 240° for grassland plant community surveys. This arrangement maximised the distance among quadrats within the same plot and allowed us to capture local-scale variation among subsampling units within relatively homogeneous meadow patches. Each meadow type included six sampling plots. At each plot, three soil samples were collected, resulting in 18 soil samples for each meadow type.

Grassland surveys and soil sampling were conducted in July 2025. To ensure representativeness, surface soil samples (0–20 cm) were collected from the center of each 1 m × 1 m quadrat after removing surface litter and roots. Soil was collected vertically using a soil shovel, thoroughly homogenized, and transferred into 50 mL centrifuge tubes, with approximately 30–40 g of soil per sample. Sample labels and sampling information were recorded on each tube. Samples were immediately stored on dry ice to minimize changes in microbial activity and biochemical processes. Each soil sample was divided into two subsamples: one was used to determine soil chemical properties and enzyme activities, and the other was stored at −80 °C for subsequent metagenomic sequencing.

### 2.3 Determination of soil chemical properties and soil enzyme activity

Soil samples were air-dried, cleared of visible impurities, and passed through a 2 mm sieve before chemical analyses. Soil pH was measured potentiometrically at a soil-to-water ratio of 1:2.5. Total nitrogen (TN) was determined using the Kjeldahl method, and hydrolysable nitrogen (HN) was measured using the alkaline hydrolysis diffusion method. Total phosphorus (TP) was determined by the molybdenum-antimony colorimetric method after nitric-perchloric acid digestion. Available phosphorus (AP) was extracted with 0.5 mol L^−1^ NaHCO_3_ (pH 8.5) and measured using the molybdenum-antimony colorimetric method. Total potassium (TK) was determined by flame photometry after nitric-perchloric acid digestion, whereas available potassium (AK) was extracted with 1 mol L^−1^ NH_4_OAc (pH 7.0) and measured by flame photometry. Soil organic matter (SOM) was determined using the potassium dichromate-sulphuric acid external heating method.

To examine differences in soil enzyme activities among sites, the activities of urease (UE), cellulase (CL), catalase (CAT), acid protease (ACPT), *β*-glucosidase (*β*-GC), and acid phosphatase (ACP) were determined using commercial assay kits (ADS series; Kangwei Century Biotechnology Co., Ltd., China) following the manufacturer’s protocols. Air-dried soil samples were passed through a 40-mesh sieve prior to analysis. The incubation temperatures were 37 °C for urease (UE), *β*-glucosidase (*β*-GC), and acid phosphatase (ACP); 40 °C for cellulase (CL); 50 °C for acid protease (ACPT); and 25 °C for catalase (CAT). After incubation, absorbance was measured using a SpectraMax Plus 384 microplate reader at wavelengths of 578 nm (UE), 540 nm (CL), 510 nm (CAT), 680 nm (ACPT), and 405 nm for both *β*-GC and ACP. Blanks and controls were included in all assays, and enzyme activities were calculated from standard curves according to the manufacturer’s instructions.

### 2.4 Soil metagenomic sequencing and bioinformatic analysis

Microbial DNA was extracted from soil samples using the E.Z.N.A.^®^ Mag-Bind Soil DNA Kit (Omega Bio-tek, USA) according to the manufacturer’s protocol. DNA concentration and integrity were assessed using a Qubit 4.0 fluorometer (Thermo Fisher Scientific, USA) and 1% agarose gel electrophoresis, respectively. For each sample, 200 ng of qualified DNA was fragmented to approximately 500 bp using a Covaris S220 ultrasonicator. Sequencing libraries were constructed using the Hieff NGS^®^ MaxUp II DNA Library Prep Kit for Illumina (Yeasen, China). After quality control and quantification using an Agilent 2100 Bioanalyzer and Qubit fluorometer, libraries were pooled in equimolar amounts and sequenced on an Illumina NovaSeq 6000 platform to generate paired-end reads (2 × 150 bp). Raw reads were quality-filtered and adapter-trimmed using fastp v0.36 (Chen et al., 2018). Host-derived contamination was removed using KneadData v0.12.0. Clean reads were assembled separately for each sample using MEGAHIT v1.2.9 (Li et al., 2015) with the parameters --k-min 21 --k-max 111 --k-step 28, and contigs longer than 300 bp were retained. Open reading frames (ORFs) longer than 100 bp were predicted using Prodigal v2.6.3. A non-redundant gene catalogue was constructed by clustering predicted genes at 95% sequence identity using CD-HIT v4.8.1. This threshold is widely used in metagenomic studies and improves analytical consistency and reproducibility (Qin et al., 2010; Ma et al., 2023). Gene abundance was estimated by mapping reads to the gene catalogue using Bowtie2 v2.1.0 and normalized as transcripts per million (TPM). Taxonomic annotation was performed using Kraken2 v2.1.3 with a confidence threshold of 0.2, in combination with Bracken against the NCBI-NT database, and was further validated using DIAMOND v2.0.13 against the NCBI-NR database (March 2023 release). Functional annotation was conducted against the KEGG database (Release 94.0) and the CAZy database (December 2022 release). Sequencing and preliminary bioinformatic analyses were conducted by Sangon Biotech (Shanghai) Co., Ltd.

### 2.5 Data analysis methods

All variables were tested for normality and homogeneity of variance before statistical analysis. Variables that did not meet the assumption of normality were log-transformed. Independent-samples *t*-tests were used to evaluate differences in plant community characteristics, soil chemical properties, enzyme activities, and microbial *α*-diversity between meadow types. Statistical analyses were performed using SPSS 27.0 (SPSS Inc., Chicago, IL, USA), with significance set at *p* < 0.05. Bar charts and boxplots were generated using GraphPad Prism 10.4.0. In R version 4.3.1, the “vegan” package (Oksanen et al., 2013) and the “ggplot2” package (Wickham et al., 2016) were used to perform principal coordinate analysis (PCoA) and permutational multivariate analysis of variance (PERMANOVA; adonis2 function in the vegan package) based on Bray–Curtis distances calculated from genus-level microbial taxonomic data to assess differences in microbial community structure between meadow types. Genus-level classification was selected because it provides a balance between ecological interpretability and statistical robustness in metagenomic datasets, reducing potential biases associated with annotation uncertainty and sparse species-level distributions while retaining meaningful ecological signals. Variance inflation factor (VIF) analysis was conducted to exclude environmental variables with VIF ≥ 10 (Kutner, 2005; Dormann et al., 2013). Redundancy analysis (RDA) was then used to identify key soil factors influencing microbial community composition and functional profiles. STAMP analysis was performed using the OmicStudio online platform^1^ to identify significantly different functional categories (*p* < 0.05). Mantel tests were conducted using Chiplot^2^ to assess correlations among soil properties, microbial communities, and plant community matrices. The “randomForest” package (Liaw and Wiener, 2002) and the “rfPermute” package (Archer and Archer, 2016) were used to evaluate the contributions of environmental factors. The 10 most abundant genera and significantly different KEGG and CAZy functional pathways were used as response variables, with soil properties used as explanatory variables. Random forest models were constructed with 999 trees, and permutation tests were applied to assess model significance and variable importance based on percentage increase in mean squared error (%IncMSE). Key drivers were visualized using heatmaps. Finally, based on the major drivers identified by multivariate analyses, partial least squares path modelling (PLS-PM) was conducted using the “plspm” package (Sanchez et al., 2013) in R to explore potential pathways influencing microbial function.

## 3 Results

### 3.1 Plant community characteristics of alpine and subalpine meadows

Quadrat surveys revealed clear habitat-related differences in plant community characteristics between the alpine meadow (AM) and subalpine meadow (SM) (Table 1). Elevation was significantly higher in AM than in SM, and vegetation cover (VC) was also significantly greater in AM, indicating pronounced differences in topographic setting and community cover between the two meadow types. No significant differences were observed in mean plant height (MH), species richness (SR), or aboveground biomass (AGB) between AM and SM (*p* > 0.05), suggesting broadly comparable community stature, richness, and productivity. However, marked differences were detected in plant functional composition and diversity patterns. Specifically, the proportion of edible forage (PEF) was significantly higher in SM, whereas the proportion of poisonous plants (PPH) was significantly higher in AM, reflecting clear divergence in plant functional composition between meadow types. In addition, the Shannon–Wiener diversity index and Pielou’s evenness index were significantly higher in SM than in AM (*p* < 0.05), indicating greater species diversity and a more even distribution of individuals in subalpine meadows. In contrast, the Margalef richness index did not differ significantly between meadow types, further suggesting that overall species richness was similar between AM and SM.

**Table 1:** Plant community characteristics in alpine meadow and subalpine meadow ecosystems.

Parameters	Alpine meadow	Subalpine meadow	Significance
Ele (m)	3912.21 ± 250.95	3271.80 ± 16.66	***
VC (%)	87.17 ± 4.02	67.33 ± 9.37	***
MH (cm)	11.29 ± 6.26	8.39 ± 1.61	ns
SR	18.00 ± 2.47	17.83 ± 3.13	ns
AGB	5821.99 ± 12949.46	1907.73 ± 467.87	ns
PEF (%)	57.95 ± 23.39	78.00 ± 13.84	**
PPH (%)	42.05 ± 23.39	22.00 ± 13.84	**
Shannon index	2.16 ± 0.24	2.34 ± 0.20	*
Pielou index	0.75 ± 0.09	0.82 ± 0.07	*
Margalef index	2.68 ± 0.39	2.86 ± 0.58	ns

Ele, elevation; VC, vegetation cover; MH, mean plant height; SR, species richness; AGB, aboveground biomass; PEF, proportion of edible forage; PPH, proportion of poisonous plants; Shannon index, Shannon–Wiener diversity index; Pielou index, Pielou’s evenness index; Margalef index, Margalef’s richness index; ns indicates *p* > 0.05; * indicates *p* < 0.05; ** indicates *p* < 0.01; *** indicates *p* < 0.001.

### 3.2 Soil chemical properties and enzyme activity characteristics of alpine and subalpine meadows

**Figure 2:**
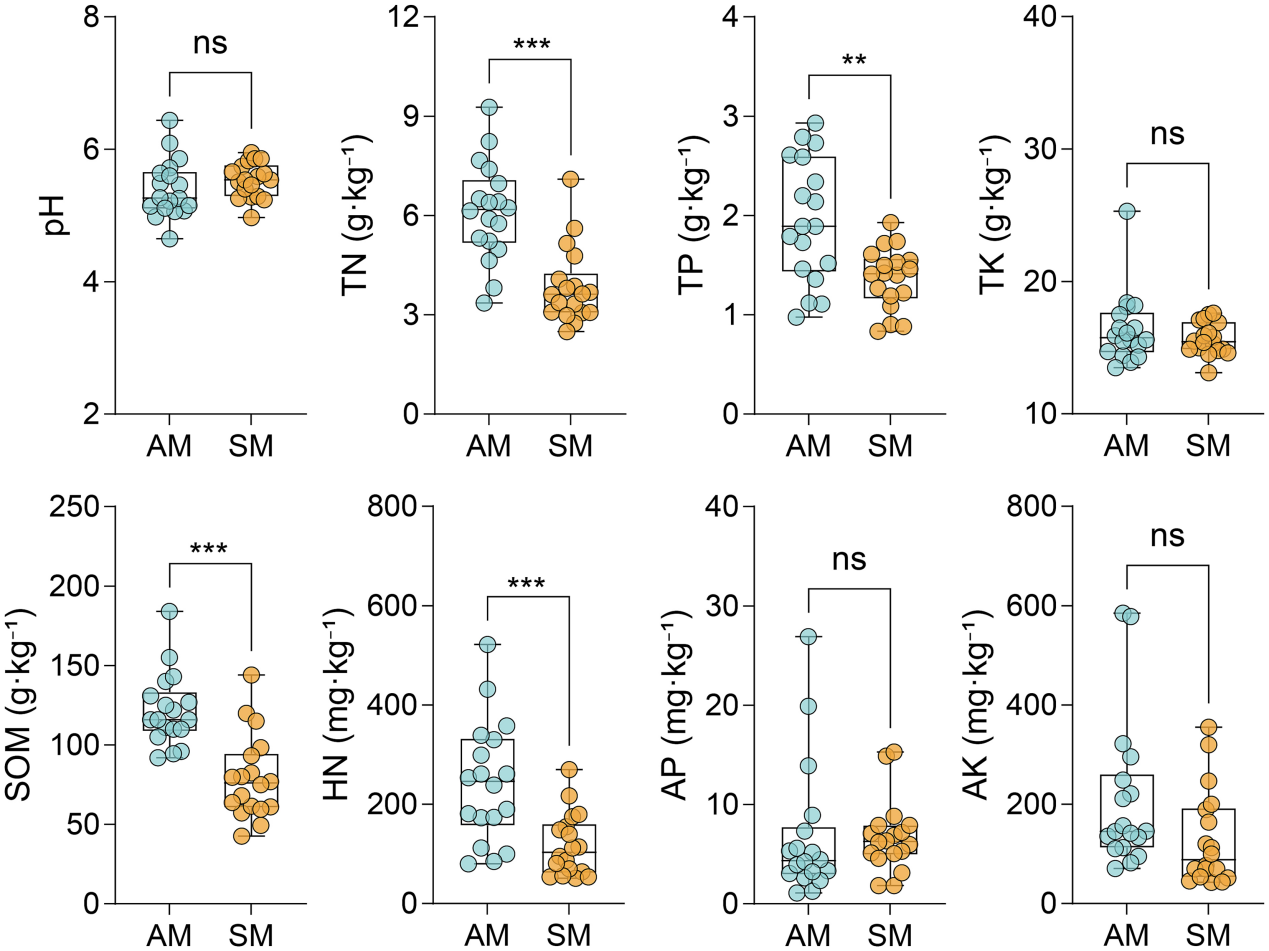
Differences in soil chemical properties between alpine meadows (AM) and subalpine meadows (SM). (1) ns indicates *p* > 0.05; ** indicates *p* < 0.01; *** indicates *p* < 0.001. (2) TN, total nitrogen; TP, total phosphorus; TK, total potassium; SOM, soil organic matter; HN, hydrolysable nitrogen; AP, available phosphorus; AK, available potassium.

Soils from alpine meadow and subalpine meadow differed significantly in chemical properties and enzyme activities. Soil chemical analyses showed that TN, SOM, HN, and TP were significantly higher in AM than in SM (*p* < 0.01; Figure 2), indicating greater accumulation of organic matter and major nutrient pools in alpine meadow soils. In contrast, AP, AK, pH, and TK did not differ significantly between the two meadow types (*p* > 0.05). These results suggest that soil nutrient differences between AM and SM were mainly associated with organic matter and nitrogen and phosphorus pools.

**Figure 3:**
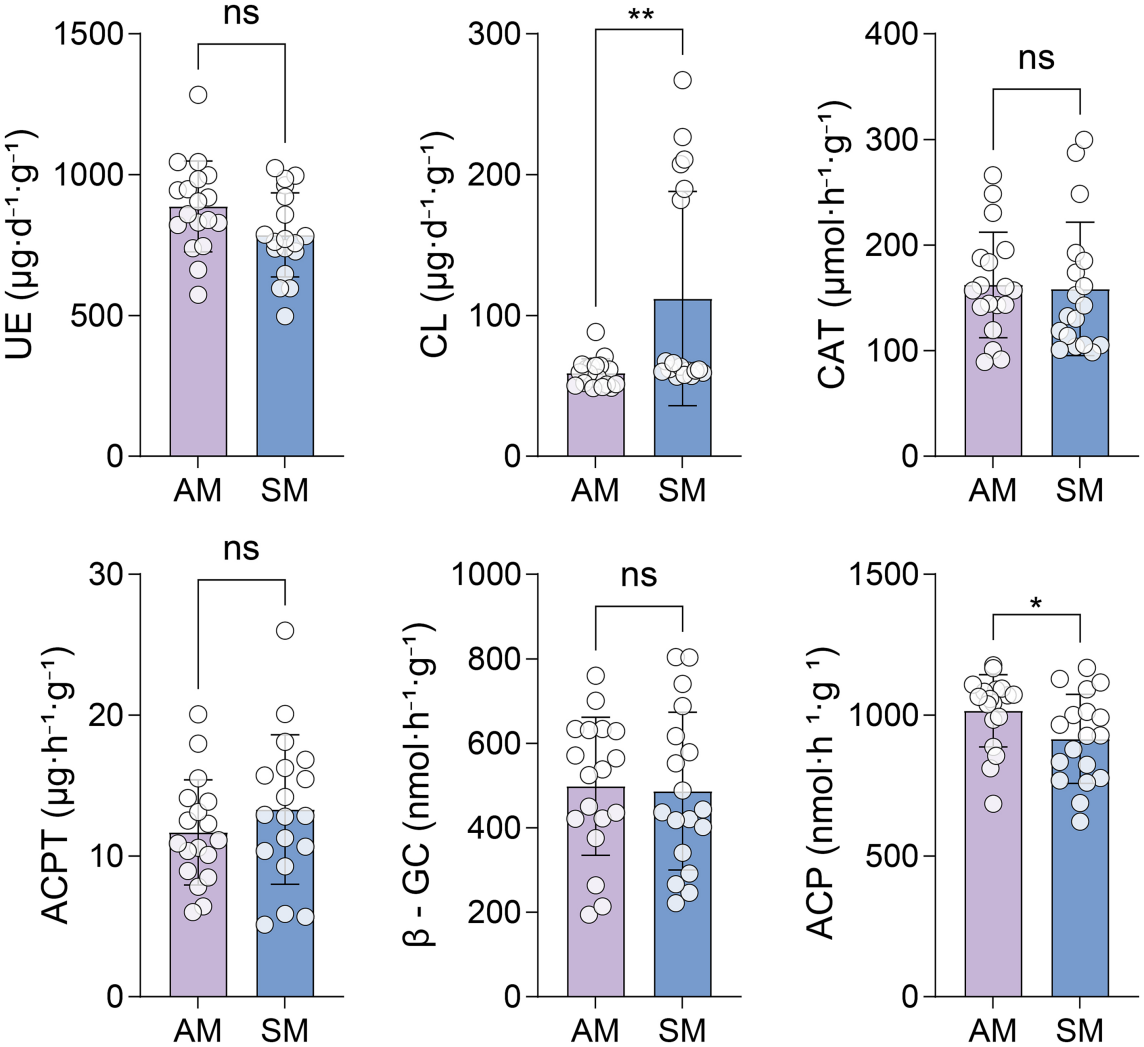
Differences in soil enzyme activities between alpine meadow (AM) and subalpine meadow (SM). (1) ns indicates *p* > 0.05; * indicates *p* < 0.05; ** indicates *p* < 0.01. (2) UE, urease; CL, cellulase activity; CAT, catalase activity; ACPT, acid protease activity; *β*-GC, *β*-glucosidase activity; ACP, acid phosphatase activity.

For soil enzyme activities, only CL and ACP differed significantly between AM and SM (*p* < 0.05; Figure 3). Other enzymes, including UE, CAT, ACPT, and *β*-GC, showed no significant differences between meadow types (*p* > 0.05). Overall, UE, ACP, *β*-GC, and CAT activities were higher in AM than in SM, whereas CL and ACPT activities were lower. This pattern indicates that soil enzyme responses to meadow type were enzyme-specific rather than uniform.

### 3.3 Metagenomic profiles of soil microbial communities and functions in alpine and subalpine meadows

**Figure 4:**
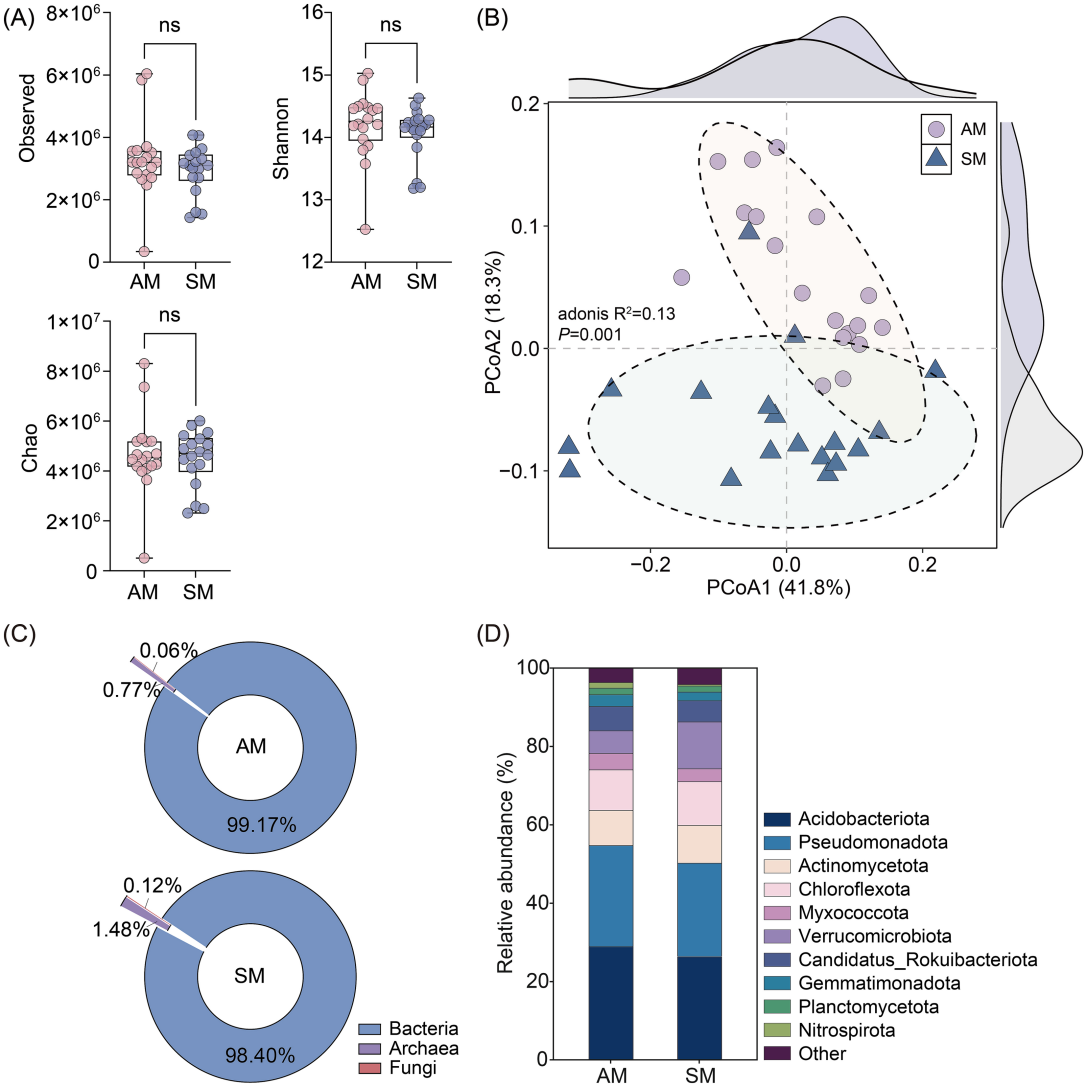
Metagenomic-based taxonomic composition of soil microbial communities in alpine meadow (AM) and subalpine meadow (SM). **(A)** Alpha diversity indices (Observed, Shannon, and Chao); **(B)** beta diversity based on Bray–Curtis dissimilarity (PCoA); **(C)** relative abundance at the domain level; **(D)** relative abundance at the phylum level.

Metagenomic taxonomic analyses showed no significant differences in microbial *α*-diversity between alpine meadow and subalpine meadow soils (*p* > 0.05; Figure 4). However, *β*-diversity analysis based on Bray–Curtis distances revealed a significant separation of microbial communities between the two meadow types (Adonis *R*^2^ = 0.13, *p* = 0.001), indicating moderate but significant differentiation in overall community structure. At the domain level, bacteria dominated microbial communities in both meadow types, accounting for more than 98% of total relative abundance. The phylum- and domain-level compositions of AM and SM were highly similar, with *Acidobacteriota*, *Pseudomonadota*, *Actinomycetota*, and *Chloroflexota* as the dominant phyla, together accounting for over 70% of total relative abundance. Significant differences were observed in the relative abundances of *Myxococcota*, *Verrucomicrobiota*, and *Nitrospirota* (Supplementary Table S1). Overall, taxonomic composition showed a relatively limited response to environmental variation, suggesting that microbial adaptation to different meadow types may occur mainly through changes in the abundance of key functional groups rather than large shifts in community membership.

**Figure 5:**
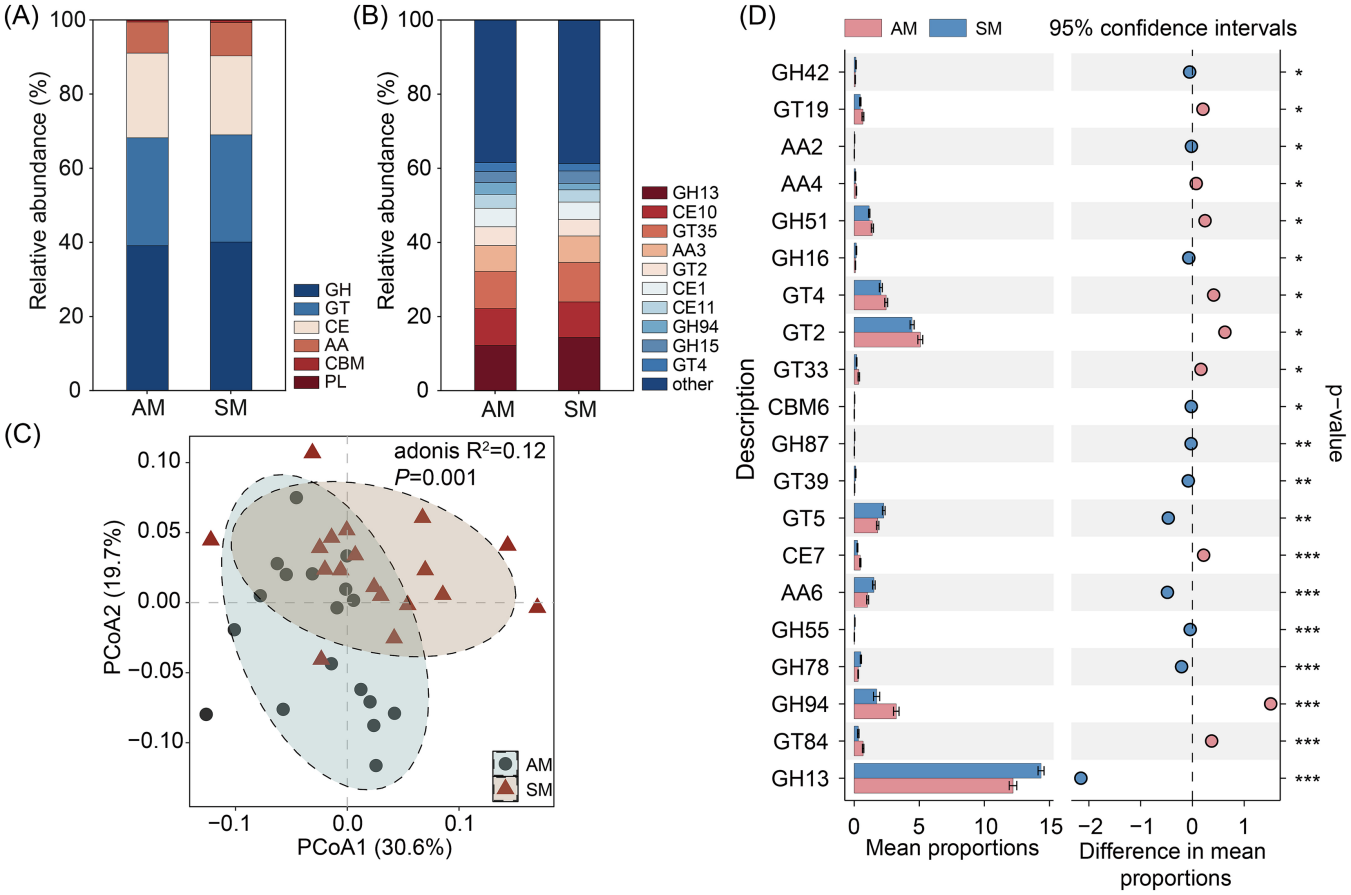
CAZyme-based functional composition and differentiation of soil microbial communities between alpine meadow (AM) and subalpine meadow (SM). **(A)** Relative abundance of carbohydrate-active enzyme (CAZyme) classes annotated against the CAZy database; **(B)** relative abundance of CAZyme families; **(C)** principal coordinates analysis (PCoA) of CAZyme functional profiles based on family-level composition (Bray–Curtis distance, Adonis test); **(D)** STAMP analysis of significantly different CAZyme families between AM and SM (Welch’s *t*-test). * indicates *p* < 0.05; ** indicates *p* < 0.01; *** indicates *p* < 0.001.

Functional annotation based on the CAZy database showed that soil microbial communities in AM and SM had broadly similar overall compositions of carbohydrate-active enzymes (CAZymes) but exhibited significant differences in functional structure and in several key families (Figure 5). At the CAZyme class level, glycoside hydrolases (GHs) dominated in both meadow types, followed by glycosyltransferases (GTs), carbohydrate esterases (CEs), and auxiliary activity enzymes (AAs). Carbohydrate-binding modules (CBMs), which act as non-catalytic domains, and polysaccharide lyases (PLs) accounted for relatively smaller proportions (Figure 5A). The similar distribution of major CAZyme classes indicates a broadly conserved framework of carbohydrate metabolism across meadow types. At the CAZyme family level, more pronounced differences emerged between AM and SM (Figure 5B). Several GH, GT, CE, and AA families differed in relative abundance, reflecting differences in microbial strategies for specific carbon source utilization and organic matter degradation. Principal coordinate analysis (PCoA) based on CAZyme family composition showed clear separation between AM and SM communities (Figure 5C). Adonis tests confirmed a significant effect of meadow type on CAZyme functional structure (*R*^2^ = 0.12, *p* = 0.001), indicating substantial functional differentiation despite overall similarity at the class level. STAMP analysis identified 20 key CAZyme families that differed significantly between AM and SM (*p* < 0.05; Figure 5D). These differences were mainly associated with GH and GT families involved in plant-derived polysaccharide degradation and carbohydrate transformation, including significant enrichment of GH13, GH94, GT5, GT2, and GT4. This pattern suggests that, although the broad carbon metabolic framework is similar, microbial communities adjust the abundance of specific CAZyme families to match differences in organic matter inputs and carbon source composition between meadow types.

**Figure 6:**
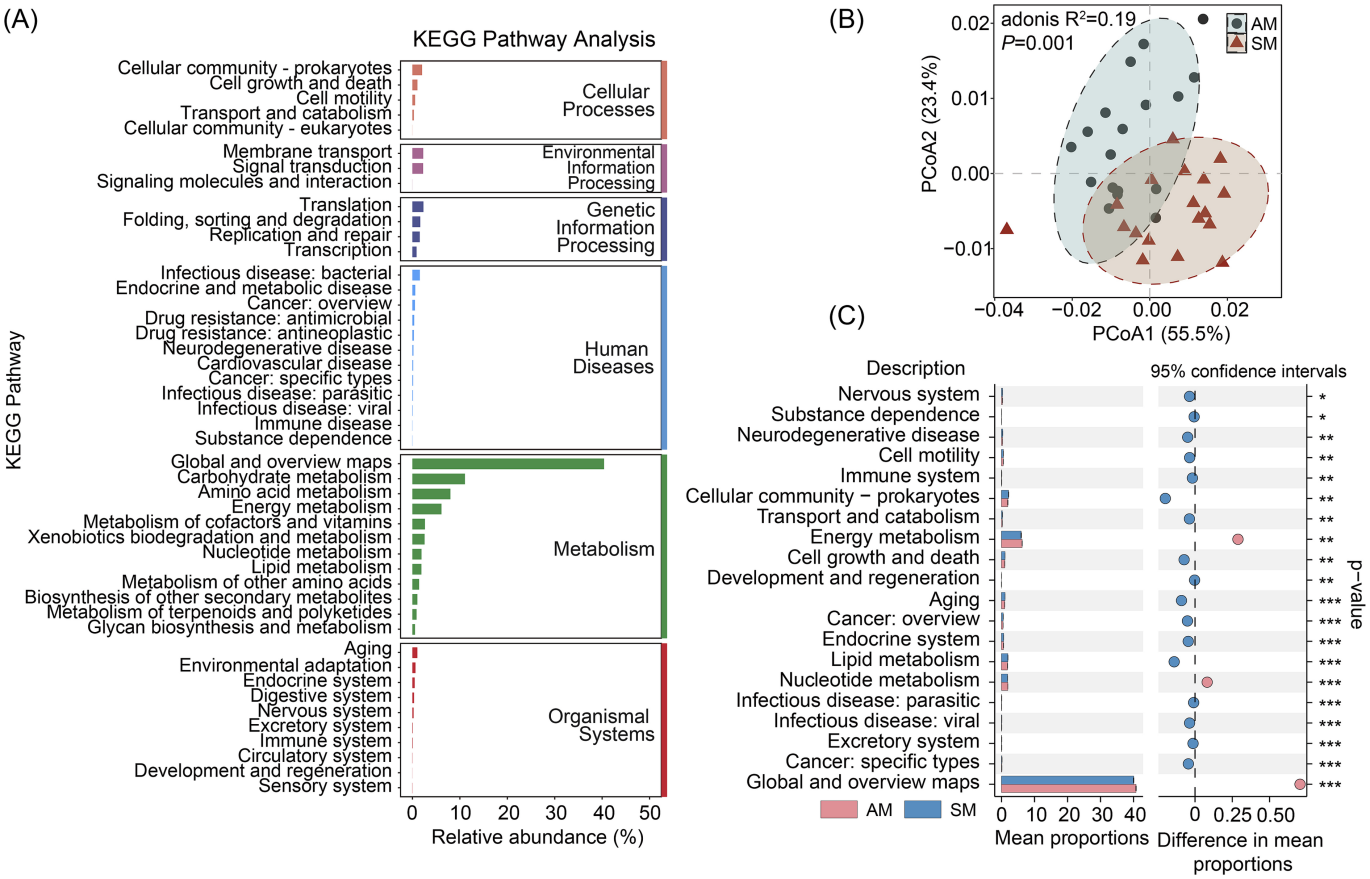
KEGG-based functional profiles and differences of soil microbial communities between alpine meadow (AM) and subalpine meadow (SM). **(A)** Relative abundance of KEGG functional pathways (Level 2); **(B)** principal coordinates analysis (PCoA) of KEGG functional profiles at Level 2 based on Bray–Curtis distance (adonis test); **(C)** STAMP analysis of significantly different KEGG pathways between AM and SM (Welch’s *t*-test). * indicates *p* < 0.05; ** indicates *p* < 0.01; *** indicates *p* < 0.001.

Metagenomic functional analysis based on KEGG pathways further showed that microbial functional profiles were broadly similar between AM and SM but differed significantly in several functional modules (Figure 6). At the KEGG level 2, metabolism-related pathways dominated in both meadow types, followed by genetic information processing, environmental information processing, and cellular processes, whereas pathways related to human diseases and organismal systems were relatively minor (Figure 6A). This indicates that soil microbial communities in both meadow types primarily support core metabolic and information-processing functions. Functional *β*-diversity analysis based on Bray–Curtis distances showed clear separation between AM and SM microbial communities (Figure 6B). Adonis tests confirmed a significant effect of meadow type on functional structure (*R*^2^ = 0.19, *p* = 0.001), indicating pronounced functional differentiation despite similar overall frameworks. STAMP analysis of KEGG level 2 pathways (Figure 6C) identified multiple pathways with significant differences between meadow types (*p* < 0.05). Pathways related to metabolism, cell motility, organismal systems, and human diseases differed in relative abundance. Notably, Global and overview maps, Energy metabolism, and Nucleotide metabolism were significantly more abundant in AM than in SM, whereas the opposite pattern was observed for several other pathways. These results suggest that soil microbial communities adapt to contrasting meadow environments by adjusting the relative abundance of key functional modules.

### 3.4 Environmental drivers of soil microbial community structure and functional in alpine and subalpine meadows

**Figure 7:**
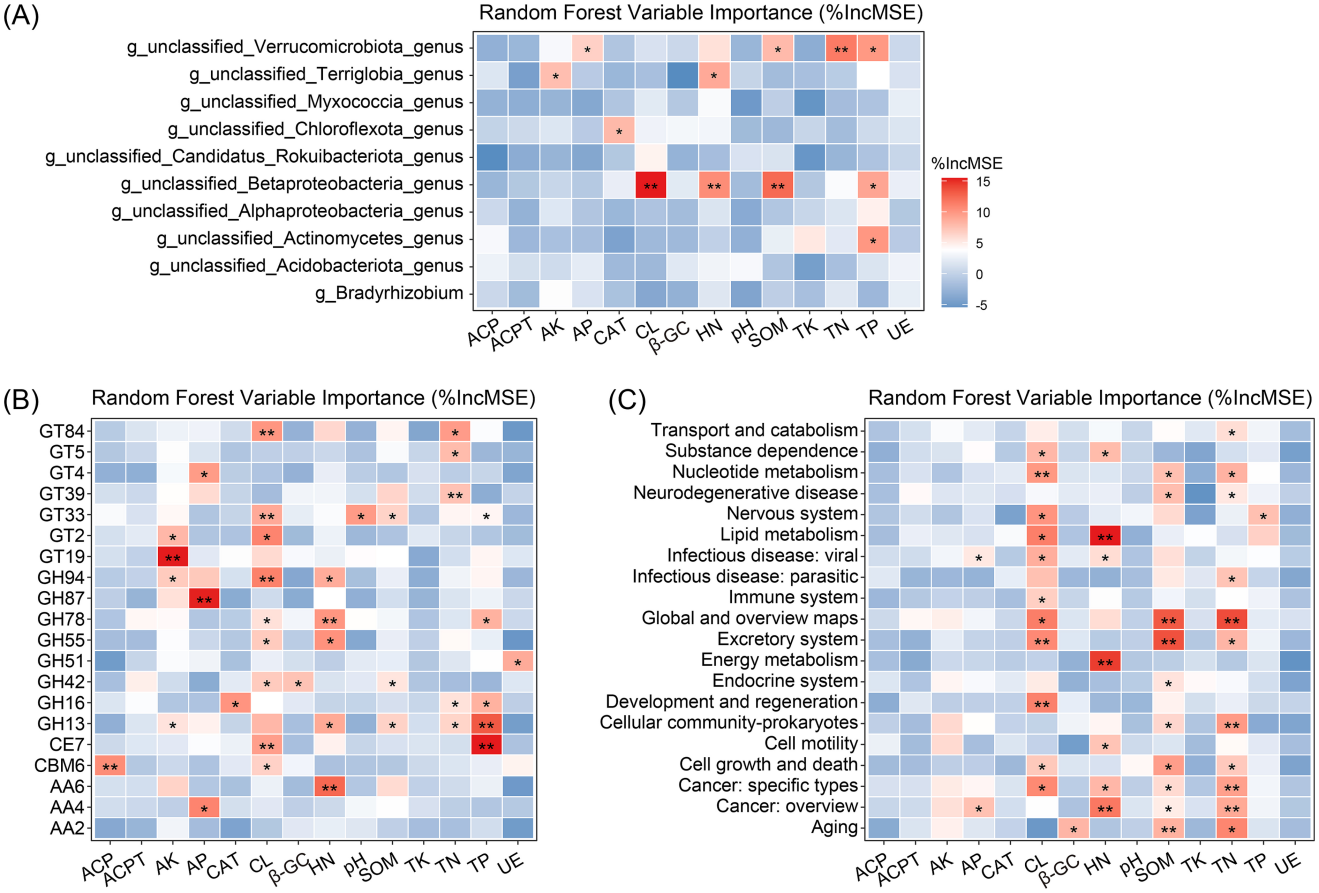
Random forest analysis identifying key soil properties driving soil microbial community structure and function in alpine and subalpine meadows. **(A)** Microbial community structure (genus level); **(B)** CAZyme functional; **(C)** KEGG pathways. Heatmaps show the relative importance of soil variables (%IncMSE), with warmer colors indicating higher importance. Asterisks denote significance (*p < 0.05, **p < 0.01, ***p < 0.001).

**Figure 8:**
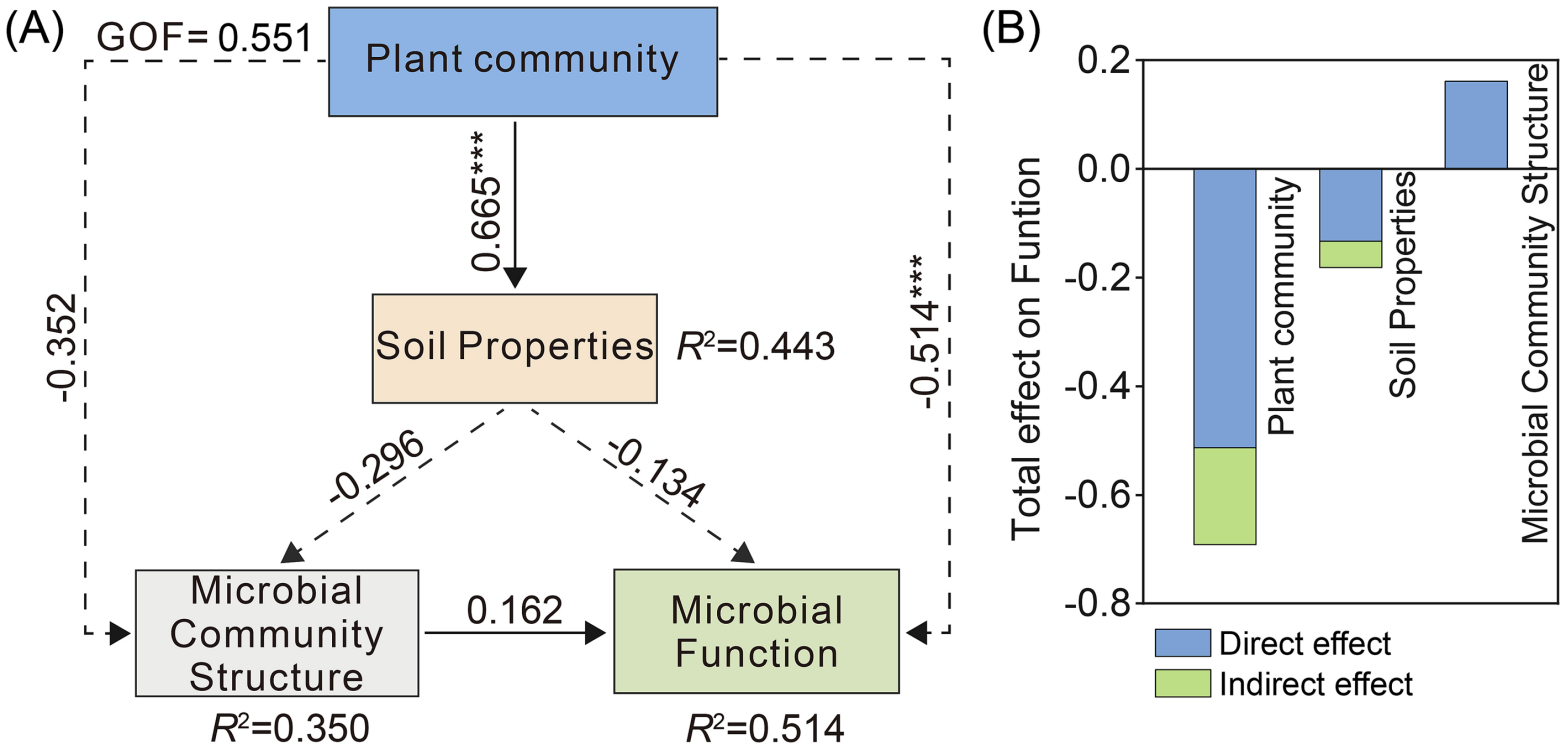
Direct and indirect effects of plant community, soil properties, and microbial community structure on microbial function revealed by partial least squares path modeling (PLS-PM). **(A)** PLS-PM illustrating the direct and indirect pathways linking plant community, soil properties, microbial community structure, and microbial function. Numbers on arrows represent standardized path coefficients, and asterisks indicate significance levels (***p < 0.001). Values adjacent to endogenous variables indicate the proportion of explained variance (*R*^2^). Model goodness-of-fit (GOF) is shown in the upper left corner. **(B)** Decomposition of the total effects of plant community, soil properties, and microbial community structure on microbial function into direct and indirect effects.

Mantel tests showed a significant association between soil properties and vegetation cover (VC) (*p* < 0.001), whereas neither microbial diversity nor microbial community structure was significantly correlated with plant community indices (*p* > 0.05; Supplementary Figure S1). These results suggest that plant community indices had limited direct associations with microbial taxonomic diversity and community structure in the study area. Based on these findings, redundancy analysis (RDA) was further used to examine the effects of soil properties on microbial community structure and functional profiles (Supplementary Figure S2). Soil environmental variables explained 42.34, 32.59, and 38.52% of the variation in microbial community structure, CAZyme profiles, and KEGG functional profiles, respectively (Figure 7), indicating that soil properties contributed substantially to microbial taxonomic and functional variation between AM and SM. Random forest analysis further identified SOM, HN, TN, TP, and CL as key factors associated with variation in microbial community structure and functional profiles in alpine and subalpine meadows. Based on these results, partial least squares path modelling (PLS-PM) was used to disentangle the pathways linking plant communities, soil properties, microbial community structure, and microbial functions (Figure 8). The overall model showed a good fit (GOF = 0.551) and explained 51.4% of the variation in microbial functions (*R*^2^ = 0.514). Path analysis showed that plant communities had a highly significant positive effect on soil properties, which in turn indirectly influenced microbial community structure and functions. Notably, plant communities also showed a significant and relatively strong direct negative effect on microbial functions. This suggests that plant communities may directly influence microbial metabolic activity and functional outputs, potentially through root exudates or other plant-mediated mechanisms, without necessarily causing detectable changes in microbial community structure.

## 4 Discussion

### 4.1 Differences in vegetation characteristics and soil properties between alpine and subalpine meadows

Our results showed significant differences in plant community characteristics and soil properties, including chemical properties and enzyme activities, between AM and SM. These differences are primarily attributable to altitude-driven variation in hydrothermal conditions (Watkins Jr et al., 2006) and are consistent with previous studies (An et al., 2020). Altitudinal gradients are widely regarded as effective proxies for species turnover because changes in elevation can markedly alter local climatic conditions (Liu M. et al., 2023), thereby influencing plant community composition. The significant difference in vegetation cover between the two meadow types was likely related to differences in dominant species and functional composition. Specifically, PEF was significantly higher in SM, whereas PPH was markedly higher in AM. Increased abundance of poisonous plants is commonly regarded as an important indicator of vegetation succession and meadow degradation, a pattern widely reported during alpine meadow degradation on the Tibetan Plateau (Li et al., 2014). Moreover, poisonous plants can persist in nutrient-poor soils and often show high tolerance to harsh environmental conditions (Hao et al., 2010). In contrast, no significant differences were observed in MH, SR, or AGB between the two meadow types. This may reflect adaptive strategies of meadow plants to temperature variation across microhabitats, enabling efficient use of solar energy for organic matter production under their respective thermal regimes (Niu et al., 2019). The Shannon–Wiener diversity index and Pielou’s evenness index were significantly higher in SM than in AM, indicating more even species distributions under relatively moderate hydrothermal conditions. By contrast, environmental stress in AM may intensify the dominance of a few tolerant species. With respect to soil properties, TN, SOM, HN, TP, and ACP were significantly higher in AM than in SM, whereas CL showed the opposite pattern. This pattern is likely associated with the high-elevation, low-temperature conditions characteristic of alpine meadows. Lower temperatures generally suppress microbially mediated organic matter decomposition and nutrient turnover, thereby favoring the accumulation of SOM, nitrogen, and phosphorus in soils (Abdalla et al., 2024). As indicators of microbial nutrient demand, soil enzyme activities are strongly influenced by temperature, soil moisture, nutrient availability, and vegetation type (Wang Z. et al., 2022; Liu S. et al., 2023). Therefore, the observed differences in CL and ACP activities between meadow types were likely linked to variation in environmental conditions and nutrient status.

### 4.2 Differential responses of microbial community structure and functional between alpine and subalpine meadows

In this study, soil microbial *α*-diversity did not differ significantly between AM and SM, whereas *β*-diversity analysis revealed significant but relatively limited community differentiation between the two meadow types (Adonis *R*^2^ = 0.13, *p* = 0.001). This suggests that microbial richness and evenness remained relatively stable across meadow types, while microbial community composition differed at the site scale, partially supporting our first hypothesis. Studies conducted on the Tibetan Plateau have similarly shown that soil microbial *α*-diversity does not necessarily change significantly along environmental gradients; however, shifts in soil physicochemical properties can markedly alter microbial community structure by regulating the relative abundances of dominant taxa, a pattern that is particularly evident in grassland degradation studies (Zhou et al., 2019). Moreover, investigations across different meadow types, including alpine swamps, meadows, and steppes, have demonstrated that meadow type per se often exerts a stronger influence on microbial community structure than on *α*-diversity (Yang et al., 2023), which is consistent with our findings. Such community differentiation was likely driven by systematic differences between meadow types in elevation, vegetation cover, litter inputs, and soil physicochemical properties, which collectively modify microbial habitats and resource availability and thereby shape soil microbial community structure (Wang et al., 2021). Across both meadow types, the dominant bacterial phyla were *Acidobacteriota*, *Pseudomonadota*, *Actinomycetota*, and *Chloroflexota*, indicating that these taxa occupy a central position in alpine meadow ecosystems. *Acidobacteriota* and *Chloroflexota* play important roles in the decomposition of complex organic matter and nutrient cycling (Eichorst et al., 2018). *Pseudomonadota* are ecologically important because of their phylogenetic diversity, functional versatility, and involvement in energy metabolism (Mukhopadhya et al., 2012), whereas *Actinomycetota* are known for their strong metabolic capacity and DNA repair mechanisms under low-temperature conditions (Johnson et al., 2007; Yergeau et al., 2010). Collectively, these metabolically active taxa with strong capacities for degrading complex organic substrates are likely to exhibit enhanced adaptability and competitive advantages under the harsh conditions of high-elevation meadows.

By integrating CAZyme profiles and KEGG pathways, we found that soil microbial communities in AM and SM shared broadly conserved functional frameworks but differed markedly in specific functional components under contrasting environmental conditions. CAZyme analysis showed that glycoside hydrolases (GHs) and glycosyltransferases (GTs) dominated in both meadow types, reflecting a general microbial capacity for plant-derived carbohydrate degradation and soil carbon cycling (Zhang et al., 2023). However, significant differences at the CAZyme family level indicate that microbial communities differed in specific carbon-degrading enzyme repertoires, likely in response to variation in organic matter inputs and carbon substrate composition between meadow types. Consistent with these patterns, KEGG-based functional analysis showed that Global and overview maps, Energy metabolism, and Nucleotide metabolism were significantly enriched in AM soils. The coupling between enhanced carbon-degrading enzyme potential and increased representation of energy- and information-related metabolic pathways suggests a coordinated functional response, whereby microbes in cold, high-stress alpine environments may sustain cellular processes through greater carbon acquisition and downstream metabolic investment (Bore et al., 2017; Feng et al., 2021). Such metabolic investment may represent an adaptive response to environmental constraints (Bore et al., 2017). In contrast, microbial communities in SM, which experience relatively higher temperatures and faster nutrient turnover, may rely more on flexible resource use and environmental responsiveness than on enhanced maintenance-related metabolic investment (Yu et al., 2025). Along the alpine–subalpine meadow gradient, soil microbial communities thus exhibit functional shifts consistent with different microbial life-history strategies, transitioning from relatively conservative, maintenance-oriented functional configurations in alpine meadows to more resource-responsive and opportunistic configurations in subalpine meadows. This functional differentiation underscores the high sensitivity of belowground carbon cycling processes and microbial metabolic organization on the Tibetan Plateau to climate-driven changes in meadow distribution.

### 4.3 Drivers of microbial functional variation

Soil microorganisms play a pivotal role in grassland succession and serve as a crucial link connecting plants, soils, and nutrient cycling (Luo et al., 2020). Previous studies have shown that soil physicochemical properties regulate microbial communities through both direct and indirect pathways, thereby influencing ecosystem productivity (Jia et al., 2020; Tong et al., 2024). Microbially mediated SOM decomposition is a key process in terrestrial biogeochemical cycles. During this process, extracellular enzymes catalyze the breakdown of complex organic substrates and release bioavailable carbon, nitrogen, and phosphorus (Tapia-Torres et al., 2015; Xu et al., 2022). Among these enzymes, CL plays an important role in plant-derived carbon degradation by hydrolyzing lignocellulosic substrates into assimilable monosaccharides, and its activity is closely associated with SOM dynamics (Wang Z. et al., 2022). The temperature sensitivity of cellulose decomposition (Fujii et al., 2023) may partly explain the higher CL activity observed in SM. In addition, HN and TN reflect soil nitrogen supply capacity and can influence microbial growth, community composition, and carbon utilization strategies (Ren et al., 2024), while TP is also essential for carbohydrate metabolism, energy carrier molecules, nucleic acid synthesis, and extracellular enzyme production, thereby enhancing microbial functional potential (Park et al., 2022; Pan and Cai, 2023).

Contrary to hypothesis (2), the PLS-PM results indicated that, within the proposed model framework, the direct pathway linking vegetation to microbial functional differentiation had a larger standardized coefficient than the soil-mediated indirect pathway. This suggests that, under the observed environmental gradients, plant community composition was more strongly associated with microbial functional differentiation than soil properties alone. Although meadow-type transitions may indirectly influence microbial community structure and function through changes in soil conditions, our model highlights the comparatively greater explanatory contribution of vegetation to variation in microbial functional potential. The use of the top 10 dominant genera and significantly differentiated metabolic pathways as response variables in the Random Forest models provides an ecologically interpretable basis for evaluating microbial responses to vegetation transitions. Dominant genera generally represent core members of the microbial community and, owing to their relatively high abundance and lower stochastic variability, are more likely to reflect stable shifts in community structure driven by environmental filtering (Wu et al., 2024; Zhang et al., 2024). In contrast, rare taxa may contribute additional noise because of their low abundance and high spatial heterogeneity. Meanwhile, differentially abundant metabolic pathways provide functional evidence for microbial adaptation to vegetation-induced environmental changes, as these pathways are closely associated with key biogeochemical processes, including carbon, nitrogen, and phosphorus cycling. Overall, this selection is consistent with our hypothesis that vegetation transitions regulate both microbial taxonomic composition and functional potential in alpine meadow ecosystems. Recent studies support this interpretation, showing that plant root-derived carbon can represent a major carbon source for soil microorganisms compared with litter-derived carbon (Feng et al., 2024). Root exudates, including amino acids, organic acids, sugars, and soluble phosphates, not only provide carbon substrates for microbial growth but also stimulate microbially mediated carbon and nutrient cycling (Sasse et al., 2018). These readily available substrates may enhance microbial carbohydrate metabolism, energy production, and nutrient acquisition pathways, consistent with KEGG categories related to carbon metabolism, amino acid metabolism, and energy metabolism. In addition, specific compounds in root exudates, such as flavonoids, organic acids, and amino acids, can influence microbial functional gene repertoires and activity (Upadhyay et al., 2022), further illustrating the potential for plant communities to modulate microbial functional potential through rhizosphere processes. Compared with carbon inputs from litter decomposition, root-derived carbon is more readily available and temporally dynamic, potentially promoting microbial investment in pathways associated with rapid resource acquisition and metabolic transformation. This plant-centered regulatory pathway suggests that microbial functional differentiation during meadow transitions is not driven solely by soil properties but is also strongly shaped by vegetation-mediated belowground inputs.

## 5 Conclusion

We systematically compared soil microbial community structure and functional potential between AM and SM and identified the key environmental factors associated with their variation. Our results showed that altitude-driven differences in hydrothermal conditions were closely linked to variation in plant community characteristics and soil properties, including nutrient status and enzyme activities, between the two meadow types. Although microbial *α*-diversity did not differ significantly between AM and SM, *β*-diversity analysis revealed pronounced differentiation in community composition, suggesting shifts in microbial community structure along the environmental gradient. Functional annotations based on KEGG and CAZyme consistently showed that variation in microbial functional potential was closely associated with the coordinated availability of carbon, nitrogen, and phosphorus, highlighting resource availability as an important factor regulating microbial metabolic strategies. Path analysis further showed that, within the hypothesized model framework, the direct effect of plant community composition on microbial functional differentiation was stronger than the indirect effect mediated by soil nutrients. This indicates that vegetation was more closely associated with variation in microbial functional potential under the current environmental gradient. Overall, this study highlights the important role of vegetation in shaping microbial functional potential in alpine-subalpine meadow ecosystems and emphasizes the sensitivity of belowground ecological processes in high-elevation meadows to environmental gradients. These findings provide new insights into the functional responses of alpine meadow ecosystems to climate change.

## Data Availability

The data presented in the study are deposited in the NCBI repository, accession number PRJNA1470787.

## Footnotes

1
https://www.omicstudio.cn/tool

2
https://www.chiplot.online
