# Supplementary material for: Relative contributions of vegetation and soil properties to microbial community structure and function in alpine and subalpine meadows of the southeastern Tibetan Plateau
Source: Front Microbiol. 2026 Jun 8;17:1847498. doi: 10.3389/fmicb.2026.1847498 (PMC13292770; doi:10.3389/fmicb.2026.1847498)
Supplement: Supplementary file 1 [file Supplementary_file_1.DOCX]

Supplementary Material

# 1 Supplementary Figures and Tables

## Supplementary Figures


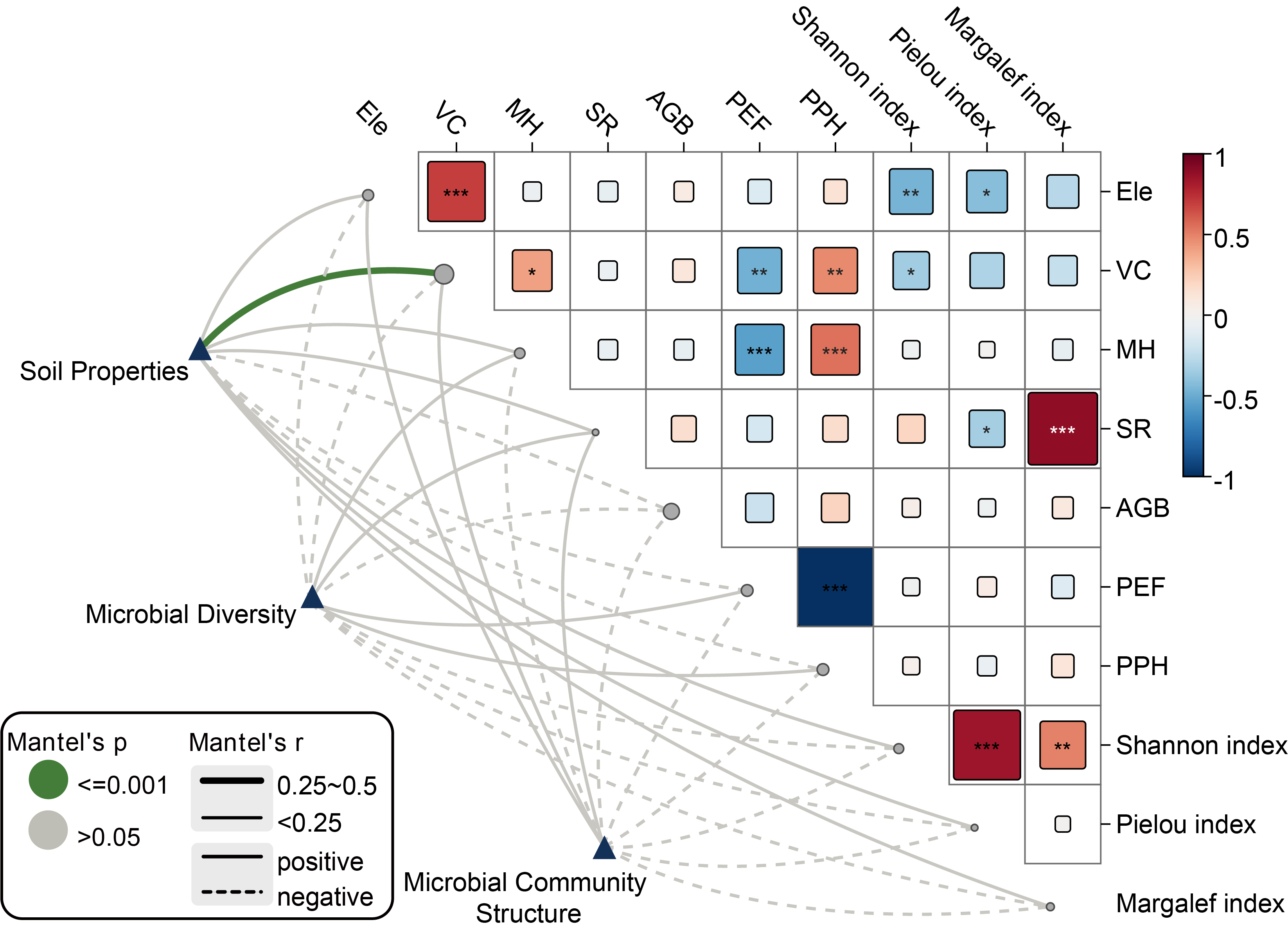


**Figure S1.** Mantel tests showing the relationships among soil properties, microbial diversity, microbial community structure, and plant community characteristics. Abbreviations: Ele, Elevation; VC, Vegetation cover; MH, Mean plant height; SR, Species richness; AGB, Aboveground biomass; PEF, Proportion of edible forage; PPH, Proportion of poisonous plants; Shannon index, Shannon–Wiener diversity index; Pielou index, Pielou’s evenness index; Margalef index, Margalef’s richness index.


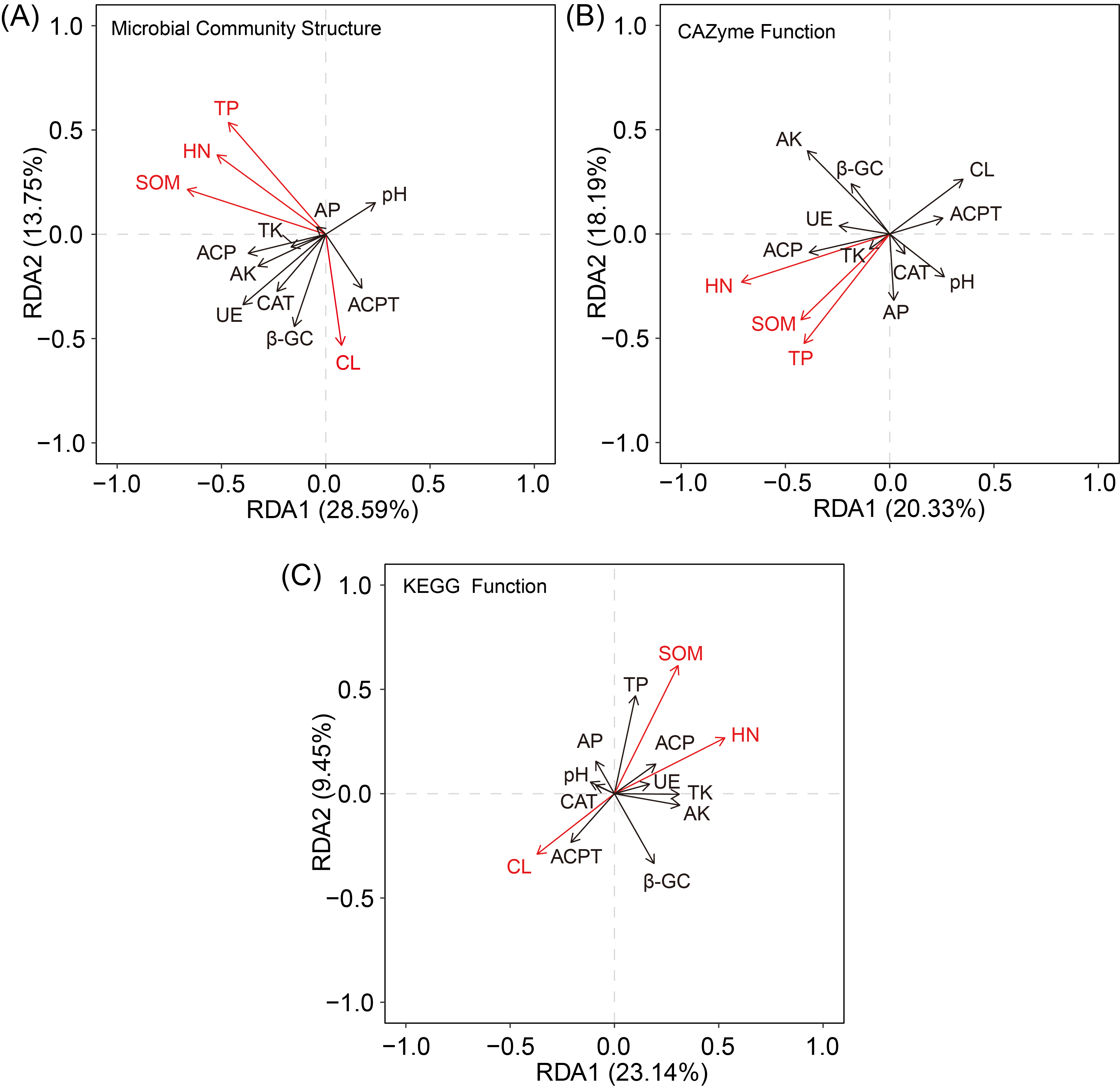


**Figure S2.** Redundancy analysis (RDA) revealing environmental drivers of soil microbial community structure and functional in alpine and subalpine meadow ecosystems. (a) Microbial community structure; (b) CAZyme functional profiles; (c) KEGG functional profiles.

## Supplementary Tables

**Table S1.** Analysis of Phylum-Level Differences in Soil Microbial Communities Between Alpine Meadows (AM) and Subalpine Meadows (SM).

| Phylum | Alpine Meadow | Subalpine Meadow | Significance |
| --- | --- | --- | --- |
| *Acidobacteriota* | 28.95±6.32 | 26.46±6.42 | ns |
| *Pseudomonadota* | 25.80±5.46 | 23.79±4.7 | ns |
| *Actinomycetota* | 8.99±2.95 | 9.66±5.84 | ns |
| *Chloroflexota* | 10.33±4.15 | 11.15±4.91 | ns |
| *Myxococcota* | 4.20±1.18 | 3.32±1.1 | * |
| *Verrucomicrobiota* | 5.78±3.04 | 11.96±5.24 | *** |
| *Candidatus_Rokuibacteriota* | 6.18±2.11 | 5.36±2.48 | ns |
| *Gemmatimonadota* | 3.01±1.38 | 2.26±0.83 | ns |
| *Planctomycetota* | 1.72±0.49 | 1.52±0.51 | ns |
| *Nitrospirota* | 1.41±0.82 | 0.36±0.64 | *** |
| *other* | 3.64±1.33 | 4.16±2.26 | ns |
| *Acidobacteriota* | 28.95±6.32 | 26.46±6.42 | ns |
